# Ani9, A Novel Potent Small-Molecule ANO1 Inhibitor with Negligible Effect on ANO2

**DOI:** 10.1371/journal.pone.0155771

**Published:** 2016-05-24

**Authors:** Yohan Seo, Ho K. Lee, Jinhong Park, Dong-kyu Jeon, Sungwoo Jo, Minjae Jo, Wan Namkung

**Affiliations:** 1 College of Pharmacy, Yonsei Institute of Pharmaceutical Sciences, Yonsei University, Incheon 406–840, Korea; 2 Department of Integrated OMICS for Biomedical Science, WCU Program of Graduate School, Yonsei University, Seoul 120–749, Korea; Albany Medical College, UNITED STATES

## Abstract

Anoctamin1 (ANO1)/transmembrane protein 16A (TMEM16A), a calcium-activated chloride channel (CaCC), is involved in many physiological functions such as fluid secretion, smooth muscle contraction, nociception and cancer progression. To date, only a few ANO1 inhibitors have been described, and these have low potency and selectivity for ANO1. Here, we performed a high-throughput screening to identify highly potent and selective small molecule inhibitors of ANO1. Three novel ANO1 inhibitors were discovered from screening of 54,400 synthetic small molecules, and they were found to fully block ANO1 channel activity with an IC_50_ < 3 μM. Electrophysiological analysis revealed that the most potent inhibitor, 2-(4-chloro-2-methylphenoxy)-N-[(2-methoxyphenyl)methylideneamino]-acetamide (Ani9), completely inhibited ANO1 chloride current with submicromolar potency. Notably, unlike previous small-molecule ANO1 inhibitors identified to date, Ani9 displayed high selectivity for ANO1 as compared to ANO2, which shares a high amino acid homology to ANO1. In addition, Ani9 did not affect the intracellular calcium signaling and CFTR chloride channel activity. Our results suggest that Ani9 may be a useful pharmacological tool for studying ANO1 and a potential development candidate for drug therapy of cancer, hypertension, pain, diarrhea and asthma.

## Introduction

ANO1/TMEM16A, a calcium-activated chloride channel (CaCC), plays an important role in fluid secretion in various cell types including airway and intestinal epithelial cells, smooth muscle cells, intestinal pacemaker cells, sensory neurons, and several tumors [1, 2]. Evidence has also been reported for ANO1 involvement in cell proliferation, cell migration, and cancer progression [3–6]. Emerging evidence suggested that pharmacological inhibition of ANO1 may be beneficial in treatment of diseases associated with ANO1 such as asthma, hypertension, diarrhea, pain and cancer. For instance, ANO1 is strongly expressed in airway mucin-secreting cells and airway smooth muscle in ovalbumin (OVA)-induced asthma mouse model. Pharmacological inhibition of ANO1 inhibits mucus secretion of airway epithelium and airway smooth muscle contraction [7]. Recently, an important role of ANO1 in the regulation of blood pressure (BP) was discovered. ANO1 is overexpressed in the arteries of spontaneously hypertensive rats (SHRs) and the treatment with T16A_inh_-A01, an inhibitor of ANO1, significantly reduced BP in SHRs, and siRNA-mediated knockdown of ANO1 prevented hypertensive development in SHRs [8]. Consistent with this finding, experimental pulmonary hypertension showed an upregulation of ANO1 on mRNA and protein levels in the pulmonary arteries of monocrotaline (MCT)-induced pulmonary hypertension rats, and 5-HT-induced contraction of pulmonary arteries significantly was reduced by pharmacological inhibition of ANO1 [9]. In rotavirus-induced diarrhea, it is considered that rotavirus nonstructural protein 4 (NSP4) acts as an enterotoxin after it is released from infected cells, activating CaCC in intestinal epithelial cells [10, 11]. It is shown that ANO1 is expressed in intestinal epithelia cells and it can be activated by NSP4_114-135_ peptide [12], and CaCC inhibition by a small molecule inhibitor can reduce rotaviral infection-induced intestinal fluid loss [13]. ANO1 plays an important role in nociception, for example, ANO1 is primarily expressed in small-diameter nociceptive dorsal root ganglion (DRG) neurons, and nocifensive behaviors are significantly reduced by pharmacological block or knockdown of ANO1 in thermal pain model [1]. In addition, capsaicin-evoked pain-related behaviors in mice are significantly reduced by inhibition of ANO1 with T16A_inh_-A01 [14]. ANO1 is amplified and highly expressed in several types of human carcinomas including head-and-neck squamous cell carcinoma (HNSCC), GIST, breast and prostate cancer. Recent evidence suggests that pharmacological inhibition of ANO1 may have beneficial effects on HNSCC, esophageal squamous cell carcinoma (ESCC), gastrointestinal stromal tumours (GIST), breast and prostate cancer [5, 15, 16]. For instance, pharmacological inhibition of ANO1 reduces cell viability in HNSCC, ESCC, breast cancer and prostate cancer cells [6, 15, 17, 18].

Thus, pharmacological inhibition of ANO1 may be beneficial in treatment of cancer, hypertension, pain, diarrhea and asthma. To date, several compounds were identified as inhibitors of ANO1 such as CaCC_inh_-A01, tannic acid, T16A_inh_-A01, digallic acid, dichlorophen, benzbromarone, N-((4-methoxy)-2-naphthyl)-5-nitroanthranilic acid (MONNA), and idebenone [7, 18–22]. However, these inhibitors have low potency and selectivity for ANO1 chloride channel.

In this study, we performed a cell based high-throughput screening for the identification of a novel potent and selective small-molecule ANO1 inhibitors. Here, we report the identification and characterization of a novel ANO1 inhibitor, which is the most potent and selective small-molecule inhibitor of human ANO1 identified to date.

## Materials and Methods

### Materials and solutions

T16A_inh_-A01, MONNA, CFTR_inh_-172, amiloride, tannic acid and other chemicals, unless otherwise indicated, were purchased from Sigma-Aldrich (St. Louis, MO). Ani9 and its analogs were purchased from ChemDiv (San Diego, CA). Recombinant Human IL-4 was purchased from R&D systems (Minneapolis, MN). The compound collections used for screening included 54,400 drug-like molecules were purchased from ChemDiv. The compounds were diluted with DMSO to reach a concentration of 2.5 mM. This was used as the 100x concentrated stock solution.

### Cell culture

Human ANO1(abc) and wild-type CFTR expressing Fisher rat thyroid (FRT) cells were prepared as described in previous study [22, 23]. ANO2 expressing FRT cells were obtained by stable transfection of FRT cells with a pCMV6-ANO2 (Origene Technologies Inc), plasmid which expresses the mouse ANO2 gene and a pcDNA3.1-YFP-F46L/H148Q/I152L plasmid which expresses halide sensor YFP gene. FRT cells cultured in Coon`s modified F12 medium supplemented with 10% FBS, 2 mM L-glutamine, 100 units/mL penicillin and 100 μg/mL streptomycin. T-84 cells were kindly provided by Dr. Min Goo Lee (Yonsei University College of Medicine, Seoul, Korea) and cultured in DMEM/F12 medium (1:1) containing 10% FBS, 100 units/ml penicillin and 100 μg/ml streptomycin. PC3 and Capan-1 cells were stably transfected with the halide sensor YFP-F46L/H148Q/I152L. The PC3 and Capan-1 cells were purchased from Korean Cell Line Bank (KCLB) and grown in RPMI 1640 medium supplemented with 10% FBS, 100 units/ml penicillin and 100 μg/ml streptomycin. Primary cultures of normal human nasal epithelial (NHNE) cells were kindly provided by Dr. Jaeyoung Choi (Yonsei University College of Medicine, Seoul, Korea). Passage-2 human nasal epithelial cells were plated at a density of 1 x10^5^ per cm^2^ onto 12-mm diameter, 0.4-μm pore polycarbonate cell culture inserts (Snapwell; Corning, Lowell, MA). The cells were maintained in 1:1 mixture of bronchial epithelial growth medium and Dulbecco’s modified Eagle’s medium containing 10% fetal bovine serum and all supplements [24]. The cells were grown at an air-liquid interface and medium was changed every 2–3 days. Cultures were used 21 days after plating at which time transepithelial resistance was 300–800 Ohm/cm^2^.

### Cell based screening

Cell based screening was performed as described in previous study [22]. Briefly, ANO1 and YFP expressing FRT cells were plated in 96-well black-walled microplates (Corning Inc., Corning, NY) at a density of 20,000 cells per well, and then cultured for 2 days. Assays were done using FLUOstar Omega microplate reader (BMG Labtech, Ortenberg, Germany) and MARS Data Analysis Software (BMG Labtech). Each well of 96-well plate was washed 3 times in PBS (200 μL/wash), leaving 100 μL PBS. Test compounds (1 μL) were added to each well at 25 μM final concentration. After 10 min, 96-well plates were transferred to a plate reader for fluorescence assay. Each well was assayed individually for TMEM16A-mediated I^-^ influx by recording fluorescence continuously (400 ms per point) for 1 s (baseline), then 100 μL of 140 mM I^-^ solution containing 200 μM ATP was added at 1 s and then YFP fluorescence was recorded for 5 s. Initial iodide influx rate was determined from the initial slope of fluorescence decrease, by nonlinear regression, following infusion of iodide with ATP.

### Short-circuit current

Snapwell inserts containing ANO1, ANO2 or CFTR expressing FRT cells, ENaC expressing T-84 cells or the human primary airway epithelial cells were mounted in Ussing chambers (Physiologic Instruments, San Diego, CA). To induce transcriptional ENaC induction, the polarized T84 cells were incubated with 2 mM sodium butyrate for 2 days. To increase the functional expression of ANO1, the human airway epithelial cells were treated with IL-4 (10 ng/mL) for 2 days. For T-84 and the human primary airway epithelial cells, the apical and basolateral bath were filled with HCO_3_^—^buffered solution containing (in mM): 120 NaCl, 5 KCl, 1 MgCl_2_, 1 CaCl_2_, 10 D-glucose, 2.5 HEPES, and 25 NaHCO_3_ (pH 7.4). For FRT cells, the basolateral bath was filled with the HCO_3_^—^buffered solution and the apical bath was filled with a half-Cl^-^ solution. In the half-Cl^-^ solution 65 mM NaCl in the HCO_3_^—^buffered solution was replaced by Na-gluconate. The basolateral membrane was permeabilized with 250 μg/mL amphotericin B. The cells were bathed for a 20 min stabilization period and aerated with 95% O_2_ / 5% CO_2_ at 37°C. ATP was applied to the apical bath solution to induce intracellular calcium increase. Ani9, T16A_inh_-A01, MONNA, amiloride, forskolin and CFTR_inh_-172 were added to the apical and basolateral bath solution. Ani9, T16A_inh_-A01 and MONNA were added 20 min before ANO1 activation. Apical membrane currents were measured with an EVC4000 Multi-Channel V/I Clamp (World Precision Instruments, Sarasota, FL) and PowerLab 4/35 (AD Instruments, Castle Hill, Australia). Data were analyzed using Labchart Pro 7 (AD Instruments). The sampling rate was 4 Hz.

### Patch-clamp

Whole-cell patch-clamp recordings were performed on ANO1-expressing FRT cells. For ANO1 currents measurement, the bath solution contained (in mM): 140 NMDG-Cl, 1 CaCl_2_, 1 MgCl_2_, 10 glucose, 10 HEPES, pH 7.4 with NaOH (310 mOsm), and the pipette solution contained (in mM): 130 CsCl, 0.5 EGTA, 1 MgCl_2_, 1 Tris-ATP, 10 HEPES, pH 7.2 with CsOH (310 mOsm). For volume-regulated anion channel (VRAC) currents measurement, the isotonic bath solution contained (in mM): 150 NaCl, 6 KCL, 1.5 CaCl_2_, 1 MgCl_2_, 10 glucose, 10 HEPES, pH 7.4 with NaOH (320 mOsm), the hypotonic bath solution contained (in mM): 105 NaCl, 6 KCL, 1.5 CaCl_2_, 1 MgCl_2_, 10 glucose, 10 HEPES, pH 7.4 with NaOH (240 mOsm), and the pipette solution contained (in mM): 40 CsCl, 100 Cs-methanesulfonate 0.5 EGTA, 1 MgCl_2_, 1.9 CaCl_2_, 5 EGTA, 4 Tris-ATP, 10 HEPES, pH 7.2 with CsOH (290 mOsm). Pipettes were pulled from borosilicate glass and had resistances of 3–5 MΩ after fire polishing. Seal resistances were between 3 and 10 GΩ. The liquid junction potential (~2.4 mV) was not corrected. After establishing the whole-cell configuration, whole-cell capacitance and series resistance were compensated with the amplifier circuitry. ANO1 was activated by ATP (100 μM). VRAC was activated by exposing LN215 cells to a 25% hypotonic bath solution. Whole-cell currents were elicited by applying hyperpolarizing and depolarizing voltage pulses from a holding potential of 0 mV to potentials between -100 mV and +100 mV in steps of 20 mV. Recordings were made at room temperature using an Axopatch-200B (Axon Instruments, Union City, CA). Currents were digitized and analyzed using a Digidata 1440A converter (Axon Instruments), and pCLAMP 10.2 software (Molecular Devices, Sunnyvale, CA). Currents were low-pass filtered at 1 kHz and sampled at 5 kHz.

### Immunoblot

FRT, FRT-ANO1, PC3, Capan-1, and NHNE cells were lysed with cell lysis buffer (50 mM Tris-HCl, pH 7.4, 1% Nonidet P-40, 0.25% sodium deoxycholate, 150 mM NaCl, 1 mM EDTA, 1 mM Na_3_VO_4_, and protease inhibitor mixture). Whole cell lysates were centrifuged at 15,000 g for 10 min at 4°C to remove the cell debris, and equal amounts (20, 80 μg protein/lane) of supernatant protein were separated by 4–12% Tris-glycine precast gel (KOMA BIOTECH, Seoul, Korea) and then transferred onto PVDF membrane (Millipore, Billerica, MA). Membrane was blocked with 5% non-fat skim milk in Tris-buffered saline (50 mM Tris-Cl, pH 7.5, 150 mM NaCl) including 0.1% Tween 20 for 1 hour at room temperature. This membrane was then incubated overnight with primary ANO1 antibody (1:500 dilution, ab64085; Abcam Inc., Cambridge, MA). After washing with 0.1% Tween 20 in Tris buffered saline (TBST), the blot was further incubated for 60 min at room temperature with an anti-rabbit secondary antibody (Santa Curz). The membrane was then washed three times with TBST for 3 minutes and then visualized using the ECL Plus western blotting detection system (GE Healthcare Amersham; Piscataway, NJ).

### Intracellular calcium measurement

FRT cells were cultured in 96-well black-walled microplates and loaded with Fluo-4 NW per the manufacturer's protocol (Invitrogen, Carlsbad, CA). Briefly, the cells were incubated with 100 μL assay buffer (1X Hanks’ balanced salt solution with 2.5 mM probenecid and 20 mM HEPES) including Fluo-4 NW. After 1 hour incubation, the test compounds were added and incubated for 20 min. The 96 well plates were then transferred to a plate reader for fluorescence assay. Fluo-4 fluorescence was measured with a FLUOstar Omega microplate reader (BMG Labtech) equipped with syringe pumps and custom Fluo-4 excitation / emission filters (485 / 538 nm). Intracellular calcium was increased by application of 100 μM ATP.

### Measurement of VRAC activity

LN215 cells, a human glioma cell line, were stably transfected with YFP-F46L/H148Q/I152L, a halide sensor YFP. After the cells were incubated on 96well black plates (Corning Inc., Corning, NY) for 48 hours, each well of the 96-well plate was washed 3 times in PBS (200 μL/wash), and the wells were filled with 50 μl/well isotonic solution (in mM): 140 NaCl, 5 KCl, 20 HEPES (310 mOsm/kg; pH 7.4 with NaOH). In each well, VRAC expressed on the cells were stimulated with addition of 50 μl of hypotonic solutions (in mM): 5 KCl, 20 HEPES, 90 mannitol (120 mOsm/kg). Test compounds (1 μL) were added to each well in a dose dependent manner. After 5 min, 96-well plates were transferred to a plate reader for fluorescence assay. Each well was assayed individually for VRAC-mediated I^-^ influx by recording fluorescence continuously (400 ms per point) for 7.6 s. YFP fluorescence was recorded 0.4 s for baseline, then 100 μL of 140 mM I^-^ solution was added at 0.4 s to see the change in fluorescence. Initial iodide influx rate was determined from the initial slope of fluorescence decrease, by nonlinear regression, following infusion of iodide.

### Statistical analysis

The results of multiple experiments are presented as the means ± S.E. Statistical analysis was performed with Student’s t-test or by analysis of variance as appropriate. A value of *P* < 0.05 was considered statistically significant.

## Results

### Identification and characterization of novel small-molecule ANO1 inhibitors

A cell-based screening of a collection of drug-like compounds was carried out to identify new classes of ANO1 inhibitors with improved potency and specificity. ANO1 chloride channel activity was measured using Fischer rat thyroid (FRT) cells stably expressing human ANO1(abc) and the genetically encoded iodide-sensing fluorescent protein, YFP-F46L/H148Q/I152L. This cell model was used because of our prior success in ANO1 chloride channel drug discovery [20, 25]. For the screening to identify ANO1 inhibitors, the FRT cells were pre-incubated with test compounds for 10 minutes prior to addition of an iodide and ATP, an agonist of P2 purinergic receptor which induces an increase in intracellular calcium concentration and activation of ANO1, containing solution. The YFP fluorescence quenching by iodide intake through ANO1 was blocked in wells containing ANO1 inhibitors.

**Fig 1:**
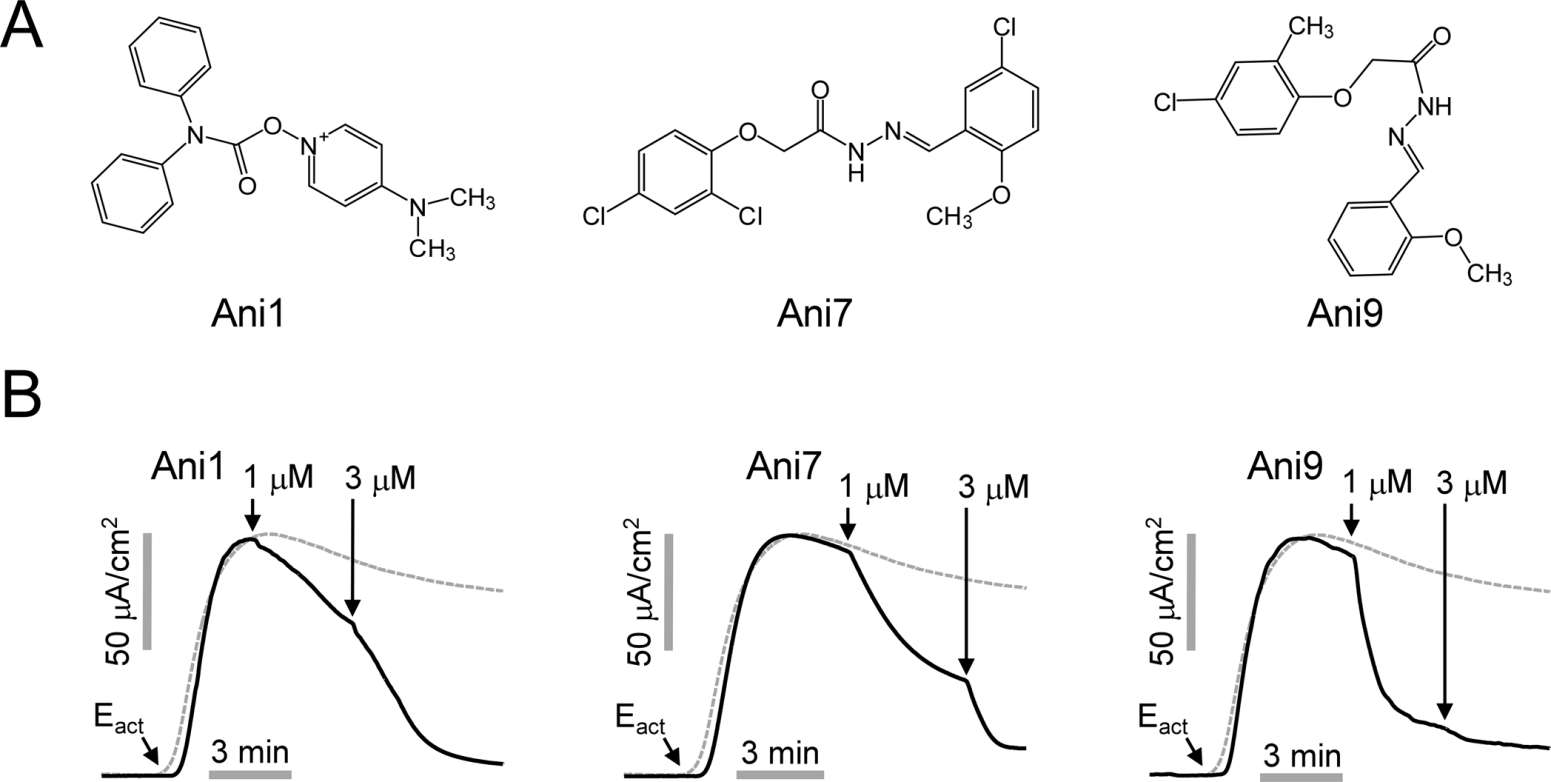
Identification of novel inhibitors of ANO1. A) Chemical structures of ANO1 inhibitors. B) Apical membrane current measured in ANO1 expressing FRT cells in the presence of a transepithelial chloride gradient. Representative current traces showing Ani1, Ani7 and Ani9-induced ANO1 inhibition at the indicated concentrations. ANO1 was activated by 10 μM E_act_, a specific activator of ANO1. The dashed gray line shows a control trace.

**Fig 2:**
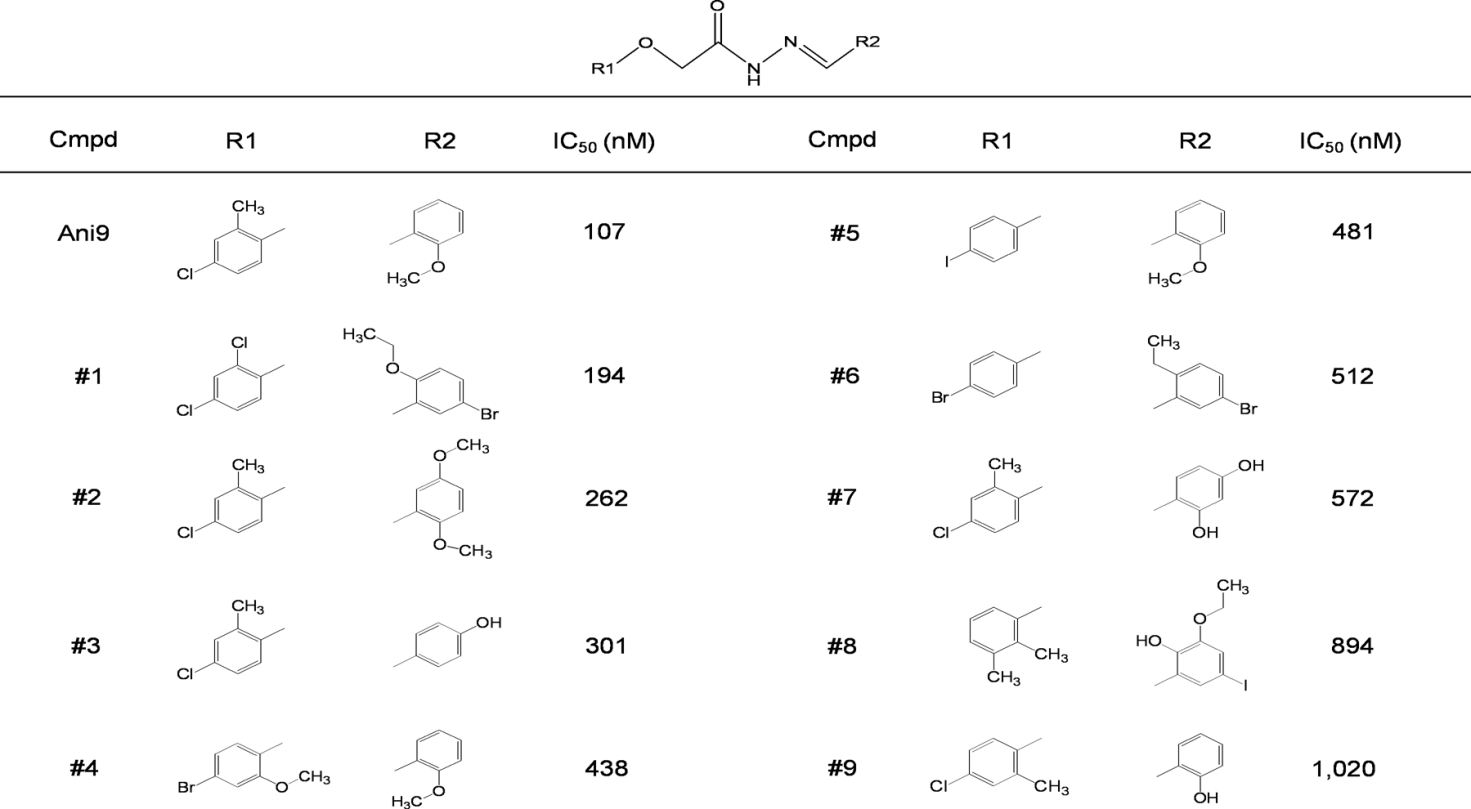
ANO1 inhibition by analogs of Ani9. Structure-activity analysis of Ani9 analogs. IC_50_ values were determined from fluorescence plate reader assay.

A total of 54,400 compounds were screened at a final concentration of 25 μM. The screening yielded 66 novel compounds that blocked iodide influx through ANO1 by > 70%. Among the hits, three most potent novel ANO1 inhibitors were selected for further investigation. Dose response studies showed that the inhibitors fully inhibited ANO1 channel activity with IC_50_ < 3 μM. The structures of the novel ANO1 inhibitors are shown in Fig 1A. To investigate the effect of the three inhibitors on ANO1 channel activity, apical membrane current was measured in FRT-ANO1 cells (Fig 1B). ANO1 was activated by 10 μM E_act_, a small-molecule ANO1 activator [25] and the small-molecule inhibitors potently inhibited the ANO1 current in a dose-dependent manner. The most potent inhibitor, Ani9, is structural analog of Ani7 and not related structurally to known ANO1 inhibitors. Further assessment of 48 commercially available Ani9 analogs showed that several compounds potently inhibited ANO1 channel activity but none were more potent than Ani9. The most active chemical analogs were summarized in Fig 2.

**Fig 3:**
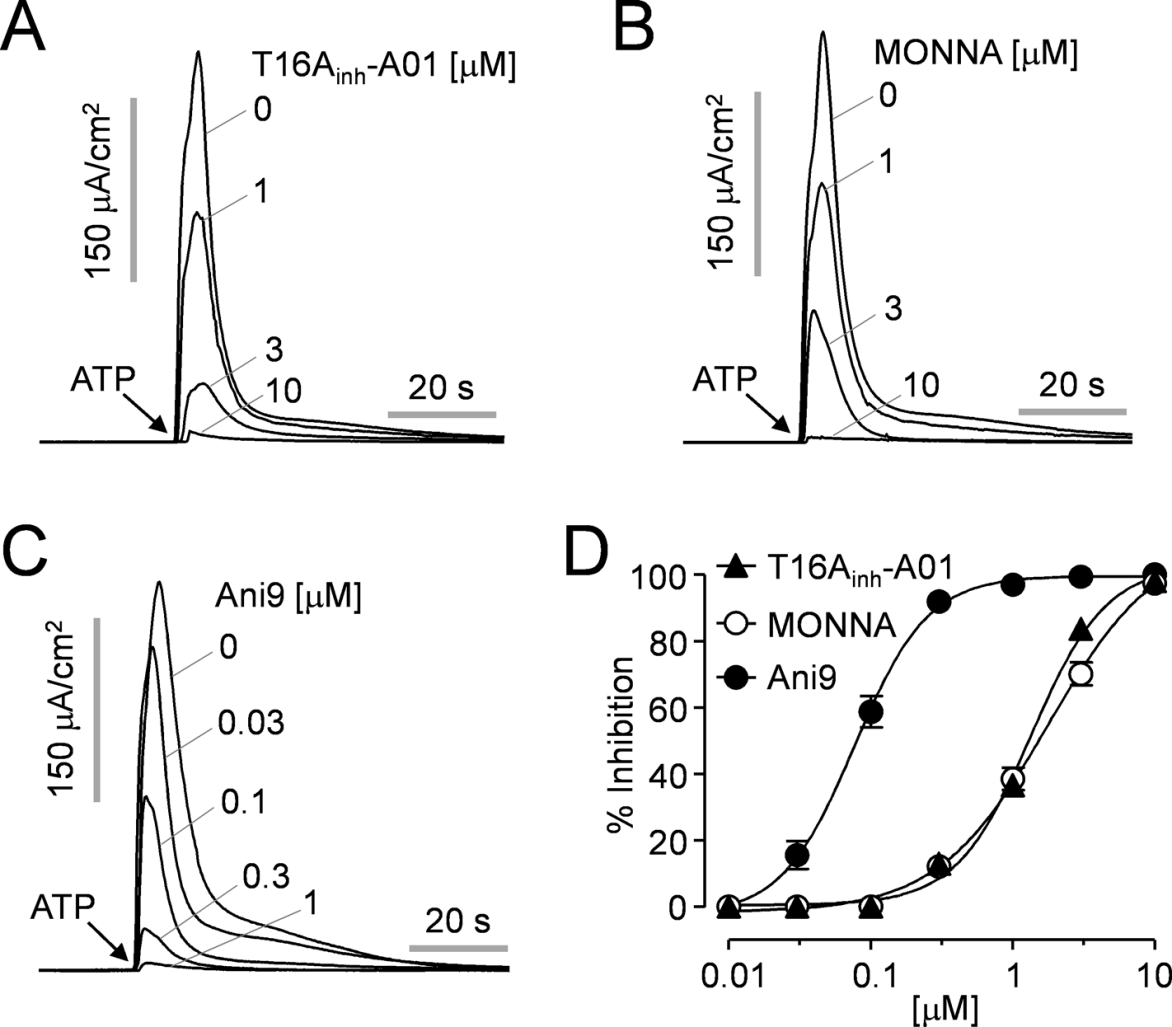
Ani9, a potent inhibitor of ANO1. A-C) Apical membrane current was measured in ANO1-expressed FRT cells. The indicated concentrations of ANO1 inhibitors, T16A_inh_-A01, MONNA and Ani9 were applied 20 min prior to ANO1 activation by 100 μM ATP. D) Summary of dose responses (mean ± S.E., n = 4–6).

To investigate the effects of the small-molecule inhibitors of ANO1 on the apical membrane chloride current, the cells were pretreated with Ani9 and the most potent known inhibitors of ANO1, T16Ainh-A01 and MONNA, for 20 min prior to ANO1 activation by ATP. Fig 3 shows apical membrane currents measurement in ANO1-FRT cell in which the cell basolateral membrane was permeabilized with amphotericin B and a transepithelial chloride gradient was applied: apical low chloride (64 mM) and basolateral high chloride (129 mM). T16A_inh_-A01, MONNA and Ani9 fully inhibited ATP-induced ANO1 activation in a dose-dependent manner with IC_50_ values of 1.39 ± 0.59 μM, 1.95 ± 1.16 μM, and 77 ± 1.1 nM, respectively (Fig 3D). Notably, the IC_50_ value of Ani9 is >18 times lower than that of T16A_inh_-A01 and MONNA.

**Fig 4:**
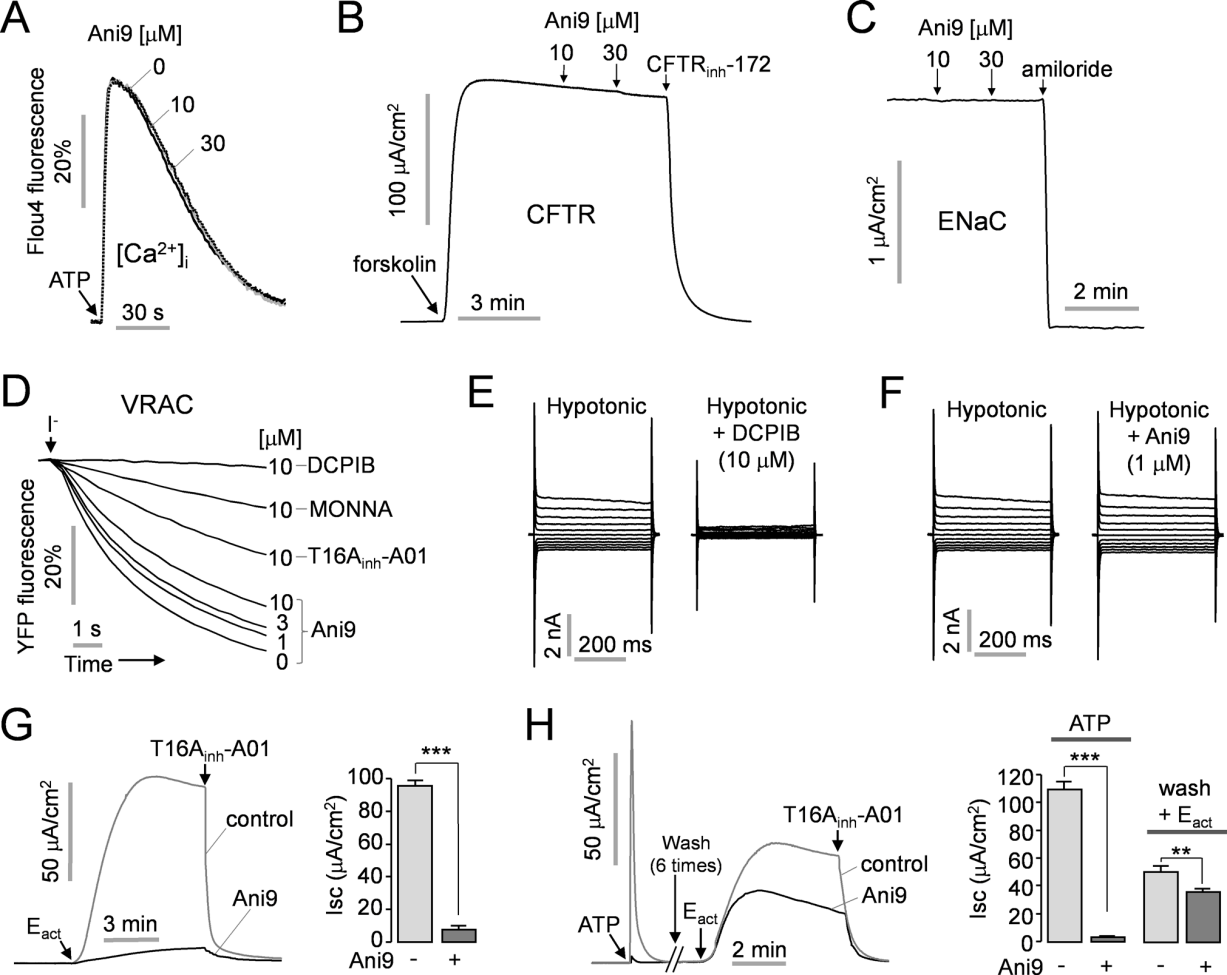
Characterization of Ani9. A) Intracellular calcium concentration was measured using Fluo-4 in FRT cells. The cells were pretreated with the indicated concentrations of Ani9 for 20 min and then 100 μM ATP, an agonist of P2Y receptor, was applied. B) Apical membrane current was measured in FRT cells expressing CFTR. CFTR was activated by 20 μM forskolin and inhibited by 10 μM CFTR_inh_-172. C) ENaC activity was measured in T84 cells. ENaC was fully blocked by 100 μM amiloride. D) VRAC activity was measured in LN215 Cells expressing endogenous VRAC and a halide sensor YFP. VRAC activity was inhibited by the indicated concentrations of ANO1 inhibitors and 10 μM DCPIB, a potent VRAC inhibitor. E, F) Whole-cell VRAC current was recorded at a holding potential at 0 mV and was pulsed to voltages between ± 100 mV (in steps of 20 mV) in the absence and presence of 10 μM DCPIB (E) or 1 μM Ani9 (F) in LN215 cells. G) Effect of Ani9 on ANO1 activation by E_act_ in FRT-ANO1 cells. 10 μM Ani9 was pretreated for 20 min and ANO1 was activated by 10 μM E_act_. The remaining ANO1 current was inhibited by 10 μM T16A_inh_-A01. (right) Summary of peak current (mean ± S.E., n = 3–4). H) Ani9 reversibility. After vanishment of 100 μM ATP-induced ANO1 current, the cells were washed six times for 5 min each and then ANO1 was activated by 10 μM E_act_. (right) Summary of peak current (mean ± S.E., n = 3–4). **P < 0.01, ***P < 0.001.

To examine whether Ani9 has an effect on cytoplasmic calcium levels, FRT cells were loaded with Fluo-4 NW, a fluorescent calcium sensor. Three separate samples of cells were pretreated with different concentrations of Ani9 (0, 10 and 30 μM) respectively for 20 min and then ATP was applied at a concentration of 100 μM to induce transient increase in cytosolic calcium (via P2Y receptor activation). Application of Ani9 up to 30 μM did not affect the ATP-induced cytosolic calcium increase in FRT cells (Fig 4A).

In order to elucidate whether Ani9 affects other ion channels including cystic fibrosis transmembrane conductance regulator (CFTR) and epithelial sodium channel (ENaC), apical membrane currents and short circuit currents were measured in FRT cells expressing human wild-type CFTR and T84 cells expressing ENaC, respectively. Fig 4B and 4C show that Ani9 had no effect on both CFTR and ENaC channel activity up to 30 μM. In addition, we observed the effect of Ani9 on volume-regulated anion channel (VRAC) in LN215, a human glioma cells, because VRAC activity could be potently blocked by CaCC inhibitors such as NS3728, T16A_inh_-A01 and NPPB [26–28]. Fig 4D shows that the previous ANO1 inhibitors, T16A_inh_-A01 and MONNA, potently inhibited VRAC activity by 71.2 ± 1.6% and 87.9 ± 1.7%, respectively, at 10 μM showing almost complete inhibition of ANO1; however, 1 μM Ani9, which showed almost complete inhibition of ANO1, inhibited VRAC activity only by 13.5 ± 1.1% and higher concentrations of Ani9 showed a relatively small inhibition of VRAC. 3 μM and 10 μM Ani9 inhibited VRAC activity by 21.4 ± 1.0% and 40.7 ± 1.5%, respectively. VRAC activity was completely blocked by 10 μM DCPIB, a selective VRAC inhibitor. To investigate the effect of Ani9 on VRAC current, we performed whole-cell patch clamp recordings in LN215 cells. The whole-cell patch clamp recordings showed that DCPIB almost completely blocked the hypotonicity-induced VRAC currents (Fig 4E). However, 1 μM Ani9, which had showed almost complete inhibition of ANO1, did not significantly affect the hypotonicity-induced VRAC currents (Fig 4F). To investigate whether Ani9 reversibly inhibits ANO1 chloride channel activity, we measured apical membrane currents in FRT-ANO1 cells. ANO1 was activated by either 10 μM E_act_, an ANO1 activator, or 100 μM ATP. Pretreatment with 10 μM Ani9 almost completely inhibited E_act_-induced ANO1 activation (Fig 4G). In Fig 4H, pretreatment with 1 μM Ani9 showed almost complete inhibition of ATP-induced ANO1 activation. However, after washing 6 times, the inhibitory effect of Ani9 on E_act_-induced ANO1 activation was significantly decreased. This result suggests that Ani9 reversibly inhibits ANO1.

**Fig 5:**
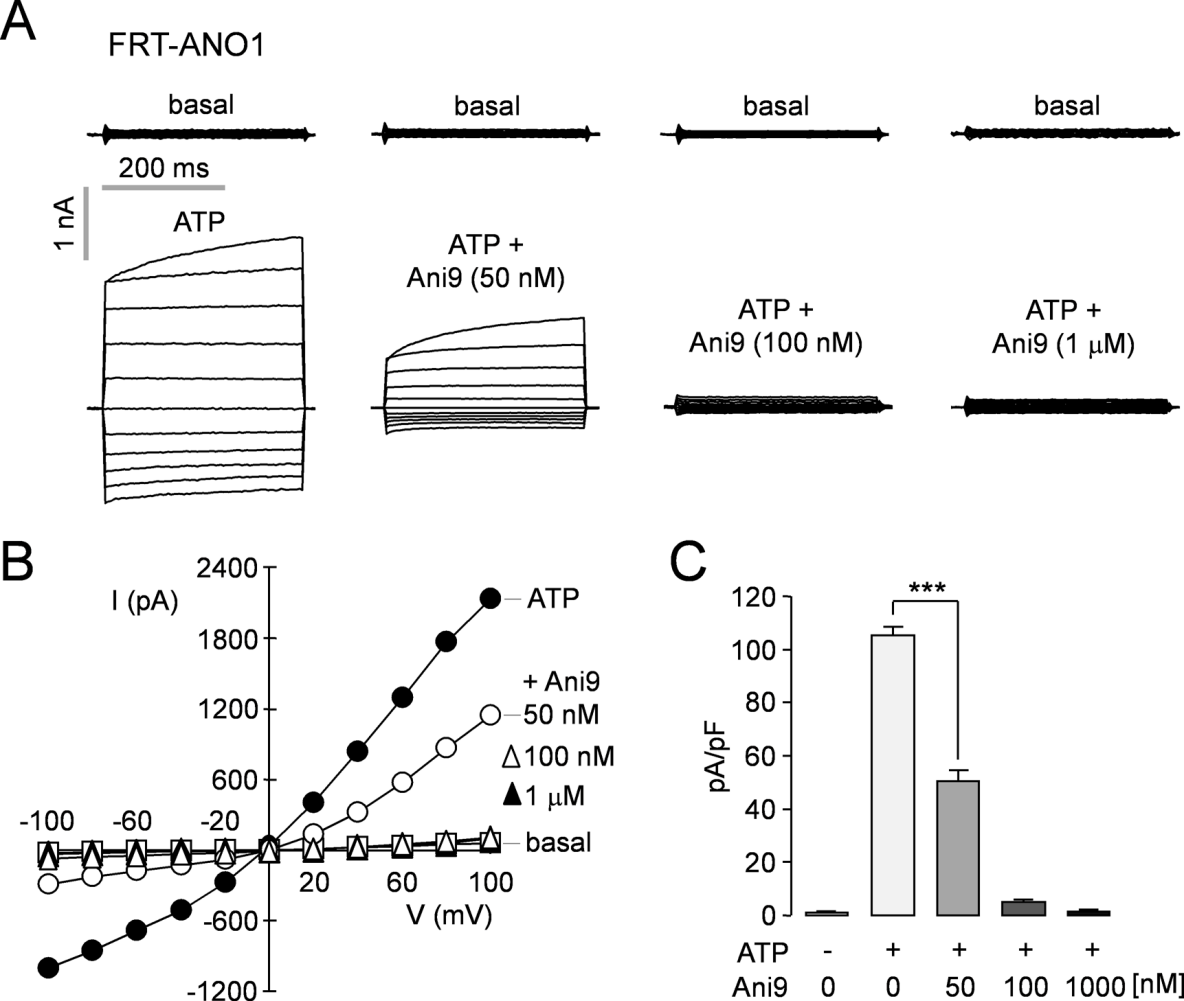
Patch-clamp analysis of ANO1 inhibition by Ani9 in FRT-ANO1 cells. A) Whole-cell ANO1 current was recorded at a holding potential at 0 mV and was pulsed to voltages between ± 100 mV (in steps of 20 mV) in the absence and presence of 50 nM, 100 nM and 1 μM Ani9. ANO1 was activated by 100 μM ATP. B) Current/voltage (I/V) plot of mean currents at the middle of each voltage pulse. C) The bar graphs summarize the current density data measured at + 80 mV (mean ± S.E., n = 4–6). ***P < 0.001.

To investigate the effect of Ani9 on ANO1 chloride channel activity, whole-cell patch clamp analysis was done in FRT cells expressing ANO1. Patch clamp measurement showed that Ani9 at 50 nM, 100 nM and 1 μM inhibits ATP-induced ANO1 chloride currents by 52.0 ± 3.7%, 95.4 ± 0.5% and 98.7 ± 0.5%, respectively (Fig 5).

### Selective inhibition of ANO1 by Ani9

**Fig 6:**
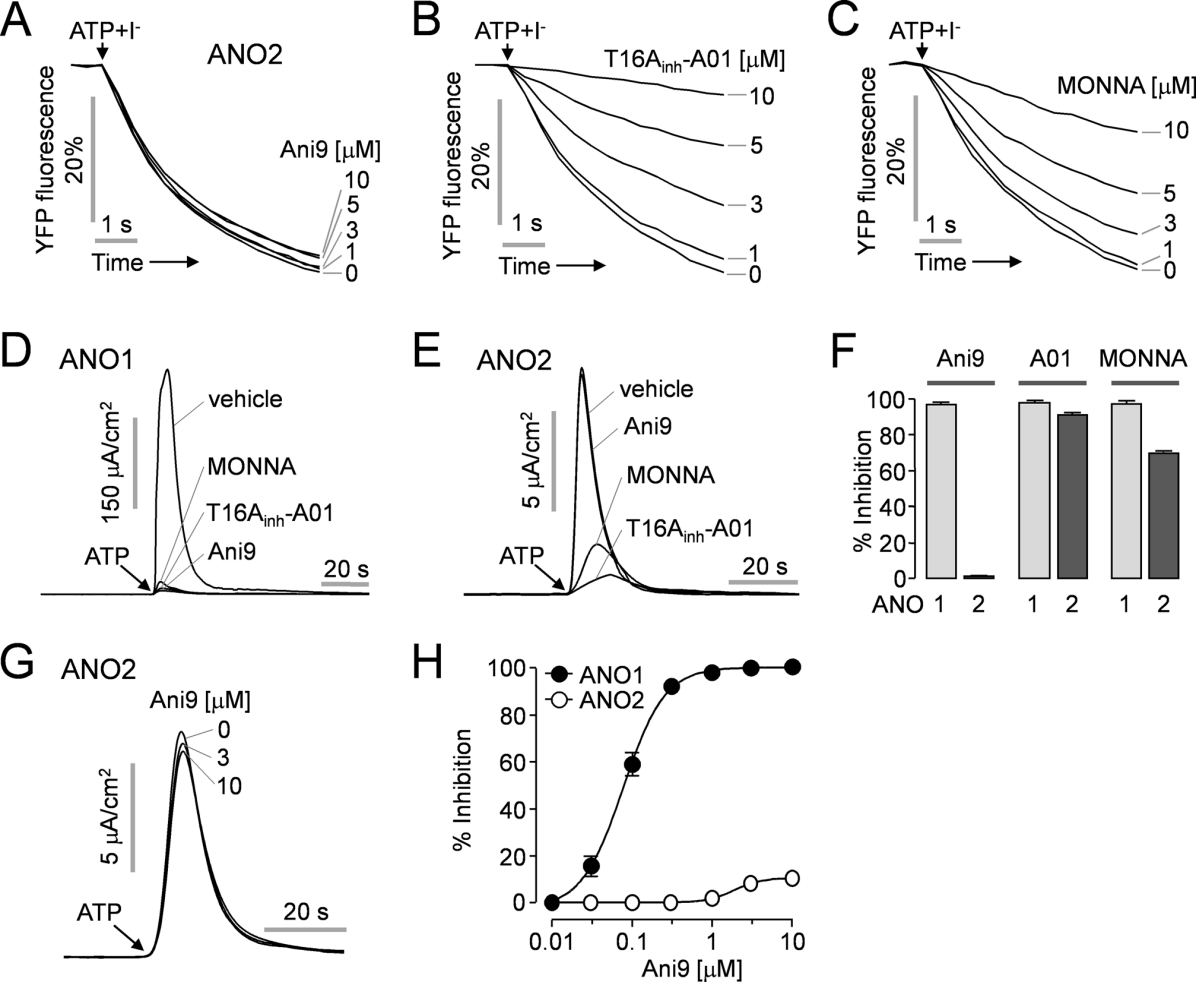
Effect of Ani9 on ANO2 activity. A-C) YFP Fluorescence measurement showing inhibition of mouse ANO2 activity by Ani9, T16A_inh_-A01 and MONNA in FRT cells expressing ANO2 and a halide sensor YFP. The indicated concentrations of the inhibitors were applied 20 min prior to ANO2 activation by 100 μM ATP. D) Apical membrane current was measured in FRT-ANO1cells. 1 μM Ani9, 10 μM T16A_inh_-A01 and 10 μM MONNA were applied 20 min prior to ANO1 activation. E) Apical membrane current was measured in FRT-ANO2 cells. 1 μM Ani9, 10 μM T16A_inh_-A01 and 10 μM MONNA were applied 20 min prior to ANO2 activation. F) Summary of peak currents of ANO1 and ANO2 (mean ± S.E., n = 4). G) Representative current traces showing Ani9-induced ANO2 inhibition at the indicated concentration. H) Summary of dose responses of ANO1 and ANO2 (mean ± S.E., n = 4–6).

ANO1 belongs to anoctamin gene family which contains ten members presenting eight-pass transmembrane segments with cytosolic N- and C-terminals. ANO2 shares a high level of amino acid homology to ANO1 (~60%) and, ANO1 and ANO2 are confirmed CaCCs. However, the roles of other family members (ANO3-ANO10) remain poorly understood and controversial [29]. To investigate whether Ani9 potently inhibits ANO2 channel activity like previous ANO1 inhibitors such as T16A_inh_-A01 [20], we measured the inhibitory effect of Ani9, T16A_inh_-A01 and MONNA on ANO2 activity using YFP quenching assay in FRT cells expressing ANO2 and the halide-sensing YFP. Ani9 did not significantly affect the ANO2 activity up to 10 μM (Fig 6A) but T16A_inh_-A01 and MONNA potently blocked the ANO2 activity in a dose dependent manner (Fig 6B and 6C). We investigated the effect of these inhibitors on ANO2-mediated apical membrane current in FRT-ANO2 cells. Pretreatment with 1 μM Ani9, 10 μM T16A_inh_-A01 and 10 μM MONNA showed almost complete inhibition of ATP-induced ANO1 current in FRT-ANO1 cells (Fig 6D). In FRT-ANO2 cells, as expected, both T16A_inh_-A01 (10 μM) and MONNA (10 μM) strongly blocked the ATP-induced ANO2 current but 1 μM Ani9 did not affect the ANO2 current (Fig 6E). The inhibition of ANO1 and ANO2 apical membrane currents by Ani9, T16A_inh_-A01 and MONNA were summarized in Fig 6F. In addition, Ani9 had very minimal effects on ANO2 apical membrane current until it reached a concentration of 10 μM. As shown in Fig 6G, pretreatment with 10 μM Ani9 only blocked the ANO2 current by 10 ± 1.6%. Fig 6H shows dose-response curves for ANO1 and ANO2. These results suggest that Ani9 is the most selective ANO1 inhibitor to date.

### Inhibition of endogenous CaCCs by Ani9

**Fig 7:**
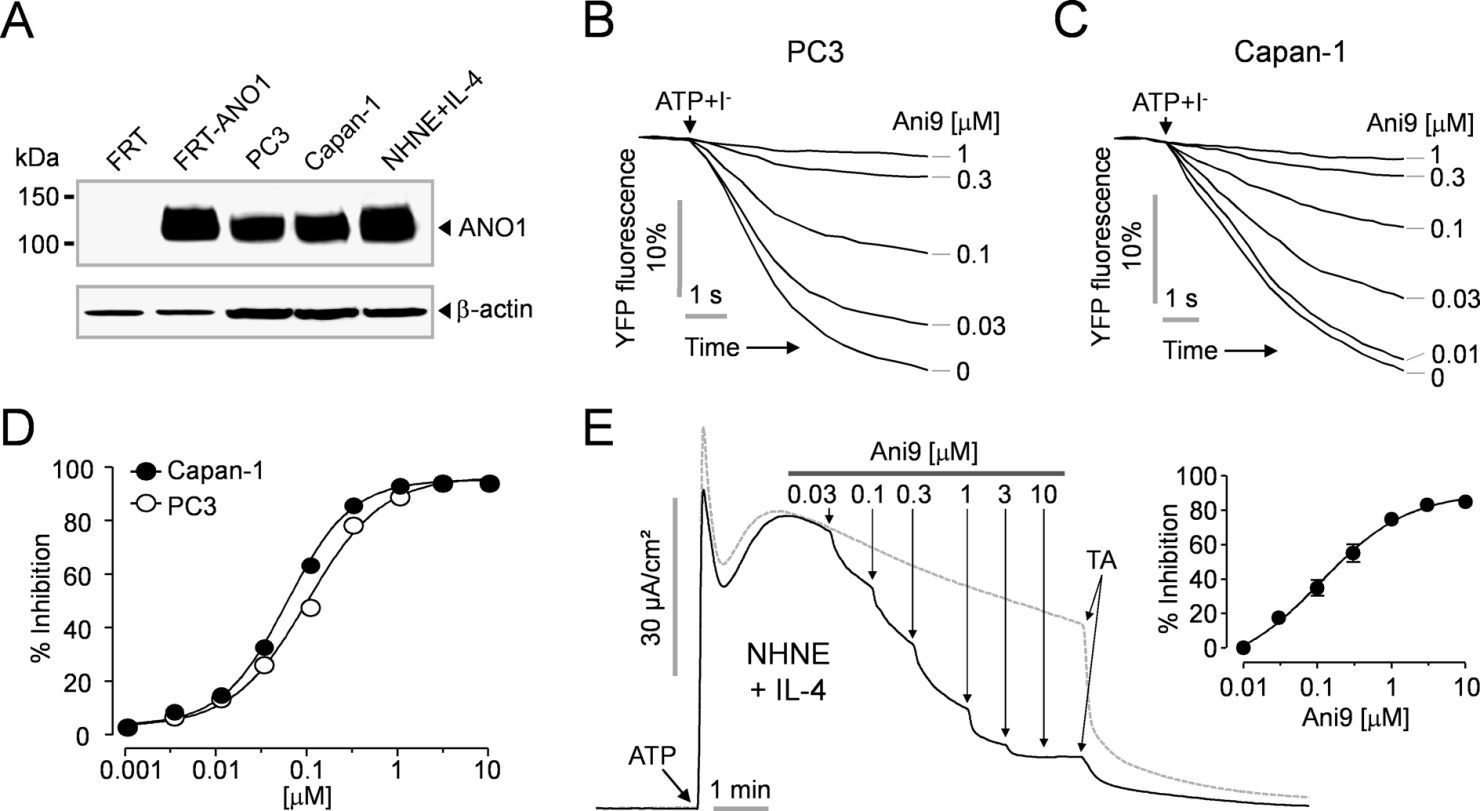
Effect of Ani9 on endogenous CaCCs in PC3, Capan-1 and NHNE cells. A) Immunoblot of ANO1 protein in FRT, FRT-ANO1, PC3, Capan-1 and NHNE cells. Blots shown are representative of experiments performed three times. B-C) Effect of Ani9 on CaCCs activity was measured in PC3 and Capan-1 cells expressing a halide sensor YFP. The indicated concentrations of Ani9 were applied 20 min prior to CaCCs activation by 100 μM ATP. D) Summary of dose response (mean ± S.E., n = 6). E) Effect of Ani9 on CaCCs activity in IL-4 treated (10 ng/mL; 48h) NHNE cells. CaCCs currents were induced by 100 μM ATP and then the indicated concentrations of Ani9 were applied. The remaining CaCCs currents were abolished by 100 μM tannic acid (TA). (right) Summary of dose response (mean ± S.E., n = 4).

To investigate the effect of Ani9 on endogenous CaCC activity, Ani9 was applied to PC3 human prostate cancer cells, Capan-1 human pancreatic carcinoma cells and primary cultured normal human nasal epithelial (NHNE) cells. As shown in Fig 7A, PC3, Capan-1 and interlukin-4 (IL-4) treated NHNE cells highly express endogenous ANO1. To measure the CaCC activity in PC3 and Capan-1 cells, the cells were stably transfected with a plasmid containing YFP-F46L/H148Q/I152L. Ani9 potently blocked ATP-induced YFP fluorescence decrease via CaCCs in both PC3 cells (Fig 7B) and Capan-1 cells (Fig 7C) in a dose-dependent manner. Fig 7D shows the inhibitory dose-response curve of Ani9 in these cells. To observe the inhibitory effect of Ani9 in primary cultured human airway epithelial cells, we measured the short-circuit current in the IL-4 treated NHNE cells because IL-4 treatment causes an increase in functional expression of ANO1 in human airway epithelium. To remove the ATP-induced CFTR activation [20] and increase the driving force for Cl^−^ secretion via CaCCs, CFTR and ENaC were inhibited by pretreatment with CFTR_inh_-172 (10 μM) and amiloride (100 μM), respectively. As shown in Fig 7E, application of ATP to the apical membrane surface triggers strong activation of CaCC and the CaCC current was significantly inhibited by Ani9 in a dose-dependent manner with IC_50_ of ~110 nM.

## Discussion

ANO1 which is a type of CaCC that can be found in various types of cells including enterocyte, DRG neuron, airway epithelial cells and vascular smooth muscle cells is overexpressed in several types of human carcinomas including head-and-neck squamous cell carcinoma (HNSCC), GIST, breast and prostate cancer [6, 13, 14, 17, 18, 30–33]. So far, some small-molecule ANO1 inhibitors such as CaCC_inh_-A01, T16A_inh_-A01, MONNA, tannic acid, benzbromarone and idebenone have been identified [18–20, 31, 32]. Previous studies have shown that inhibition of ANO1 by small-molecule inhibitors reduce cancer cell proliferation [32], rotavirus-induced diarrhea [13], capsaicin-induced nocifensive behaviors [14, 30], and contractile tone in vascular and airway smooth muscle [31, 33, 34]. Therefore, ANO1 inhibitors may be useful for the treatment of cancer, diarrhea, pain, hypertension and asthma.

In this study, we found highly potent and selective ANO1 inhibitor, Ani9, from a screening of 54,400 small molecules because all the known inhibitors have low potency or low selectivity against ANO1. As shown in Fig 3D, measurement of apical membrane current in FRT-ANO1 cells revealed that the novel ANO1 inhibitor Ani9 has an IC_50_ of 77 ± 1.1 nM, which is >18 times more potent than T16A_inh_-A01 and MONNA IC_50_ of 1.39 ± 0.59 μM and 1.95 ± 1.16 μM, respectively.

ANO2 has homology to ANO1 with 62% amino acid identity within the ANO Family [2]. Unlike other members of anoctamin family, ANO1 and ANO2 function as endogenous CaCCs in many cell types [35–37]. ANO1 and ANO2 share same properties as CaCCs including anion selectivity, submicromolar sensitivity to intracellular calcium and voltage activation at low calcium concentrations, and it is known that both ANO1 and ANO2 can be inhibited by nonspecific CaCC inhibitors such as niflumic acid (NFA) and 5-nitro-2-(3-phenylpropylamino)-benzoic acid (NPPB) [38]. In Fig 6, we showed that the previous best ANO1 inhibitors, T16A_inh_-A01 and MONNA, potently inhibit ANO2 activity in a dose-dependent manner. However, 1 μM Ani9 causing almost complete inhibition of ANO1 did not affect ANO2 channel activity, and higher concentrations (3 and 10 μM) of Ani9 showed a relatively small inhibitory effect on ANO2. These results indicate that Ani9 can be a useful pharmacological tool for the selective isolation of ANO1 from endogenous CaCCs in multiple cell types expressing both ANO1 and ANO2.

Previous studies have shown that a number of CaCC inhibitors potently block VRAC activity [26, 28]. Thus there is a possibility that Ani9 also has an inhibitory effect on VRAC activity. In this study, we elucidated the effect of the potent ANO1 inhibitors, Ani9, T16A_inh_-A01 and MONNA, on VRAC activity using YFP quenching assay in LN215 cells expressing endogenous VRAC and an YFP-based halide sensor (Fig 4D). Of interest, 1 μM Ani9 inducing almost complete inhibition of ANO1 hardly affected VRAC activity even though T16A_inh_-A01 and MONNA had strongly inhibited VRAC activity at a concentration of 10 μM which caused almost complete inhibition of ANO1. In addition, our study showed that Ani9 does not affect other ion channels such as CFTR and ENaC at a high concentration (Fig 4B and 4C). Together, the above results indicate that Ani9, a novel ANO1 inhibitor, is the most potent and selective human ANO1 inhibitor to date.

A recent report indicates that ANO1 is involved in the development of benign prostatic hyperplasia (BPH), and pharmacological inhibition of ANO1 inhibits prostate enlargement and reduces histological abnormalities in the BPH rat model [39]. In addition, ANO1 inhibitors inhibit cell proliferation of prostate and pancreatic cancer cells [4, 40], and reduce mucus production in primary human airway epithelium and contraction of airway smooth muscle [7]. Thus, ANO1 inhibitors may be useful for the treatment of BPH, prostate and pancreatic cancer, and inflammatory airway diseases. To investigate the inhibitory effect of Ani9 on endogenous CaCCs, we observed the inhibition of endogenous CaCCs activity by Ani9 in PC3 human prostate cancer cells, Capan-1 human pancreatic cancer cells and IL-4 treated NHNE cells because PC3, Capan-1 and IL-4 treated human primary airway epithelial cells highly express endogenous ANO1 [40–42]. As expected, Ani9 very potently inhibited endogenous CaCCs activity in all three cell types although the potencies of Ani9 were slightly different in these cells (Fig 7). The slight difference in Ani9 potency may be due to the difference of the membrane permeability of Ani9 and the contribution of ANO1 to total CaCCs in these cell types. These results suggest that Ani9 or its derivatives can be a viable potential candidate for further investigation on the development of drug therapy for above diseases.

In summary, to the best of our knowledge, Ani9 represents the most potent and selective small-molecule inhibitor of human ANO1 with IC_50_ ~77 nM. Ani9 causes almost complete inhibition of ANO1 at a concentration of 1 μM but does not affect ANO2 activity at the same concentration. In addition, it did not affect the other ion channels including CFTR, VRAC and ENaC at a high concentration required for the full inhibition of ANO1. Thus, the ANO1 inhibitor identified here constitutes new and readily accessible chemical tool for pharmacological dissection of ANO1 and may be a potential development candidate for drug therapy of cancer, hypertension, pain, diarrhea, asthma and other ANO1 related diseases.
